# Self-Phosphorylated Polybenzimidazole: An Environmentally Friendly and Economical Approach for Hydrogen/Air High-Temperature Polymer-Electrolyte Membrane Fuel Cells

**DOI:** 10.3390/membranes13060552

**Published:** 2023-05-25

**Authors:** Igor I. Ponomarev, Dmitry Y. Razorenov, Kirill M. Skupov, Ivan I. Ponomarev, Yulia A. Volkova, Konstantin A. Lyssenko, Anna A. Lysova, Elizaveta S. Vtyurina, Mikhail I. Buzin, Zinaida S. Klemenkova

**Affiliations:** 1 A.N. Nesmeyanov Institute of Organoelement Compounds of Russian Academy of Sciences, Vavilova St., 28, Bld. 1, 119334 Moscow, Russia; razar@ineos.ac.ru (D.Y.R.); ivan.ponomarev84@gmail.com (I.I.P.); yvolk@ineos.ac.ru (Y.A.V.); ves1809@yandex.ru (E.S.V.); buzin@ineos.ac.ru (M.I.B.); zkeem@ineos.ac.ru (Z.S.K.); 2 Faculty of Chemistry, Lomonosov Moscow State University, GSP-1, Leninskie Gory, 1-3, 119991 Moscow, Russia; klyssenko@gmail.com; 3 Kurnakov Institute of General and Inorganic Chemistry, Leninskii Prosp., 31, 119071 Moscow, Russia; ailyina@yandex.ru

**Keywords:** polybenzimidazole, polyamide, proton-conducting membrane, polymer-electrolyte membrane, fuel cell, HT-PEM, phosphorylation, proton conductivity, membrane-electrode assembly, pre-polymer

## Abstract

The development of phosphorylated polybenzimidazoles (PBI) for high-temperature polymer–electrolyte membrane (HT-PEM) fuel cells is a challenge and can lead to a significant increase in the efficiency and long-term operability of fuel cells of this type. In this work, high molecular weight film-forming pre-polymers based on *N^1^*,*N^5^*-bis(3-methoxyphenyl)-1,2,4,5-benzenetetramine and [1,1′-biphenyl]-4,4′-dicarbonyl dichloride were obtained by polyamidation at room temperature for the first time. During thermal cyclization at 330–370 °C, such polyamides form *N*-methoxyphenyl substituted polybenzimidazoles for use as a proton-conducting membrane after doping by phosphoric acid for H_2_/air HT-PEM fuel cells. During operation in a membrane electrode assembly at 160–180 °C, PBI self-phosphorylation occurs due to the substitution of methoxy-groups. As a result, proton conductivity increases sharply, reaching 100 mS/cm. At the same time, the current-voltage characteristics of the fuel cell significantly exceed the power indicators of the commercial BASF Celtec^®^ P1000 MEA. The achieved peak power is 680 mW/cm^2^ at 180 °C. The developed approach to the creation of effective self-phosphorylating PBI membranes can significantly reduce their cost and ensure the environmental friendliness of their production.

## 1. Introduction

Polybenzimidazoles (PBIs) are heterocyclic polymers based on aromatic tetramines and dicarboxylic acids. They present one of the most important types of high-performance heterocyclic polymers [1]. PBIs are widely used for the fabrication of fibers, films, plastics, and, in particular, solid polymer proton-conducting membranes for high-temperature polymer electrolyte membranes (HT-PEM) imbibed (doped) in phosphoric acid. Currently, there is an urgent need to replace traditional fossil fuels with renewable energy sources such as hydrogen [2,3]. Hydrogen HT-PEM fuel cell (HT-PEMFC), operating at 150–200 °C, can produce electricity and heat for several thousand hours even under such extreme conditions [4]. The main advantage of the PBI membrane-based HT-PEMFC [2,5,6,7] is the ability to work with hydrogen contaminated with CO; it is different from low-temperature PEMFC (LT-PEMFC) [8] with Nafion^®^ membranes [9,10] which require hydrogen of high purity. The most studied PBI is the commercially available poly-2,2′-(*m*-phenylene)-5,5′-bibenzimidazole (*m*-PBI, Celazole^®^) based on isophthalic acid and carcinogenic 3,3′-diaminobenzidine (DAB) created by Vogel and Marvel in 1961 [11]. A further history of the *m*-PBI can be found on PBI Performance Products, Inc. website [12]. The industrial production of *m*-PBI consists of a two-stage melt polycondensation of DAB with diphenyl isophthalate [13]: 1 h at 260 °C in an inert atmosphere and 9 h at 260–400 °C under vacuum. Such a polymer contains a gel fraction, and its molecular weight (M_w_) does not exceed 40 kDa. In order to use this polymer for membrane preparation, it should be fractionated and reprecipitated from *N*,*N*-dimethylacetamide (DMAc) solution.

Synthesis of PBIs from basic monomers, i.e., aromatic tetraamines and dicarboxylic acids, in polyphosphoric acid (PPA), known as the Iwakura method [14], is well known as the most commonly used method to prepare a large number of different PBIs. Here PPA is used as a solvent and condensation agent for the efficient removal of water released during the polycondensation. Due to the advantages of moderate temperatures (170–230 °C) and the use of more stable tetraamine monomers in the form of tetrahydrochloride, this approach is an excellent universal route for the preparation of high-molecular-weight PBIs. The main drawback is a quite low polymer concentration (3–5 wt%) in the PPA solution since PBI isolation is associated with an enormous amount of acid waste.

**Figure 1:**
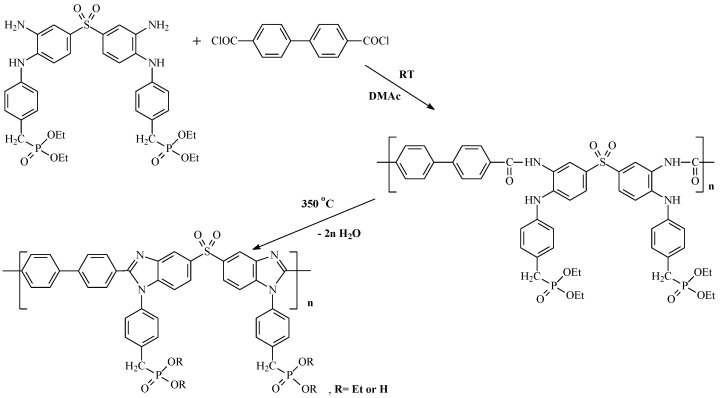
Two-step synthesis of phosphorylated PBI-SO_2_-P.

Kim et al. used a mixture of methane sulfonic acid—P_2_O_5_ (9:1 *w*/*w*) to synthesize many PBIs at rather low temperatures (120–140 °C) [15]. Recently, we have proposed a new approach to phosphorylated polybenzimidazole two-stage synthesis via polyamidation and subsequent thermal cyclization [16] (Figure 1).

The reaction in the DMAc medium proceeds smoothly for several hours at room temperature and a total monomer concentration of 35 wt.% with the formation of a high molecular weight polymer. According to gel permeation chromatography (GPC), the molecular weight reaches 340 kDa and the polydispersity index (PDI) is 1.85. Strong and elastic films (Young’s modulus, E, 1600–1800 MPa; tensile strength, σ, 90–110 MPa; elongation at break, ε, 10–15%) were cast from reaction solutions and subjected to thermal heterocyclization in an inert medium at 250–370 °C for 2 h to form phosphorylated PBI-SO_2_-P functionalized by phosphonate groups. This environmentally friendly process eliminates huge acid runoff and energy costs related to classical PBI production processes. In the presence of monomers with phosphonate groups, this process allows the synthesis of polymers with high proton conductivity and high resistance to phosphoric acid leaching from a proton-conducting membrane during HT-PEM fuel cell operation [16].

Phosphoric acid leaching during HT-PEM fuel cell operation is a problem, which can affect the long-term durability of PBI-based cells and stacks. According to [17], the nitrogen atoms of benzimidazole are fully protonated with a low rate of proton exchange with phosphate species; so that, the processes of proton diffusion and conduction should occur in the hydrogen bond network of phosphoric acid only. The proton exchange dynamics between the phosphate and benzimidazole species pass through an intermediate exchange mode with the exchange times being close to the typical diffusion times selected in the pulsed-field gradient nuclear magnetic resonance (PFG-NMR) diffusion measurements. The loss of phosphoric acid destroys the hydrogen bond network, resulting in reduced proton transfer efficiency. Therefore, phosphorylation of PBI should result in the stabilization of the hydrogen bond network and proton transport through the membrane due to phosphonate groups linked to the main chain of the polymer.

The presence of phosphate groups in the main chain of PBI can also reduce the migration of the phosphoric acid electrolyte from the membrane to the anode in HT-PEMFC during fuel cell operation at 160–200 °C. Acid migration is driven by a non-zero transference number for the hydrophosphate anion. The acid migration rate in the range 0.2–0.8 A/cm^2^ shows a sudden increase at current densities of 0.4–0.6 A/cm^2^ (the most important operating range of current densities for HT-PEMFC) [18]. 

Phosphonated polymers are attractive systems for several reasons. First of all, they exhibit efficient proton transport under low water uptake conditions. This is related to a higher degree of hydrogen bonding which promotes the proton transport by the Grotthuss mechanism [19]. Second, the phosphonic acid group can release two protons instead of one, because organophosphonates are dibasic compared with organosulfonates which are monobasic. Third, phosphonated polymers often show higher chemical and thermal stability compared with sulfonic acid moieties, in part, due to their higher pK_a_ values [20]. 

In spite of these advantages, relatively few studies of phosphonated polymers have been reported [21]. This is likely because of a very narrow set of synthetic routes to phosphonated polymers [20,21,22,23,24,25]. In fact, almost all research on proton transport in phosphonated polymer systems is limited. While for such copolymers, the proton conductivity range 10^−6^–10^−1^ S/cm has been reported.

In our previous works [26,27,28,29,30,31], we have explored the PBI polymer chemistry and membrane composite materials to increase their proton conductivity and reduce phosphoric acid leaching.

**Figure 2:**
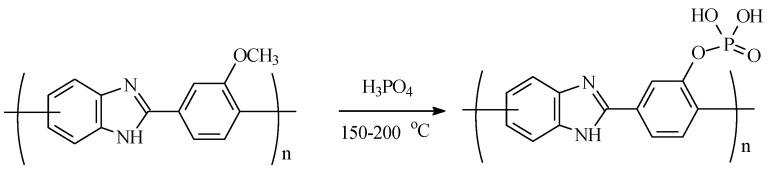
Phosphorylation reaction scheme.

It should be noted that phosphorylated monomers and polymers based on them are very expensive and difficult to obtain synthetically by default. Therefore, the search for alternative ways to obtain them is relevant and extremely demanded. It was found that the demethylation of aryl methyl ethers in a mixture of phosphorus pentoxide in methanesulfonic acid occurs in a phosphoric acid medium [32]. Based on these data, it was assumed that in the presence of methoxy-groups in the PBI chain, when operating in a fuel cell on a PBI membrane doped with phosphoric acid, the formation of self-phosphorylated membranes with a complex of improved properties is possible due to demethylation. A simplified scheme of the phosphorylation reaction is shown in Figure 2.

The current study aimed to obtain a more efficient phosphorylated PBI membrane for HT-PEM fuel cell applications. 

## 2. Materials and Methods

### 2.1. Materials

All chemicals, 1,5-dichloro-2,4-dinitrobenzene, *m*-anisidine, [1,1′-biphenyl]-4,4′-dicarbonyl dichloride, *N*,*N*-dimethylacetamide, *N*-methylpyrrolidone (NMP), hydrazine hydrate, Pd/C (10%), phosphoric acid (85%), were obtained from Acros Organics (part of Thermo Fisher Scientific, Waltham, MA, USA) and used as received without additional purification.

### 2.2. Gel Permeation Chromatography

The molecular weights of samples were measured by gel permeation chromatography using Agilent 1200 gel permeation chromatography (Agilent Technologies, Santa Clara, CA, USA) with a refractometric detector and PLmix C (5 mm) column. 0.03 M LiCl solution in *N*-methylpyrrolidone was used as eluent at 25 °C with a flow rate of 0.5 mL/min. Calibration was performed using polystyrene standards.

### 2.3. Thermogravimetric Analysis

Thermogravimetric analysis (TGA) measurements were performed in air using a Derivatograph-C (MOM Szerviz, Budapest, Hungary) at a heating rate of 5 K/min. Sample weight taken ~12 mg. 

### 2.4. Differential Scanning Calorimetry

Differential scanning calorimetry (DSC) was performed using a differential scanning calorimeter DSC-3 (Mettler Toledo, Greifensee, Switzerland) at a heating rate of 10 K/min.

### 2.5. Nuclear Magnetic Resonance

Nuclear magnetic resonance (NMR) spectra of the studied compounds were recorded on a Bruker Avance 400 spectrometer (Bruker, Billerica, MA, USA).

### 2.6. Fourier-Transform Infrared Spectroscopy (FTIR)

Fourier-transform infrared spectroscopy (FTIR) of individual compounds (in KBr pellets) and polymer films was performed in absorbance mode using an InfraRed Bruker Tensor 37 FTIR spectrometer (Bruker, Billerica, MA, USA) with the spectral range 7500–370 cm⁻^1^ and resolution of <0.6 cm⁻^1^.

### 2.7. Elemental Analysis

The elemental composition was determined with an Elementar Vario MICRO cube C,H,N-analyzer (Langenselbold, Germany) equipped with a thermal desorption column and spectrophotometrically using a Cary 100 Scan UV-Vis spectrophotometer (Agilent Technologies, Santa Clara, CA, USA) after Schoniger method combustion (for F) and after decomposition by concentrated sulfuric acid as heteropoly acid according to Kjeldahl method (for P).

### 2.8. X-ray Analysis

Single crystals of (**3**) (C_36_H_24_F_6_N_4_O_2_, M 658.59) were investigated on a Bruker D8 QUEST single-crystal X-ray diffractometer (Bruker, Billerica, MA, USA) equipped with PHOTONII detector, charge-integrating pixel array detector (CPAD), laterally graded multilayer (Goebel) mirror and microfocus Mo-target X-ray tube (λ = 0.73071 Å). Data reduction and integration were performed with the Bruker software package SAINT, version 8.40B (Bruker, Billerica, MA, USA). At 119 K, crystals of **3** are triclinic, space group P-1, Z = 2 (Z’ = 1), a = 10.6021 (12) Å, b = 10.6271 (11) Å and c = 15.1993 (15) Å, α = 72.446 (3)°, β = 82.584 (4)°, γ = 60.933 (3)°, V = 1426.8 (3) Å^3^. A total of 13,936 reflections (2θ_max_ = 58°, R_int_ = 0.0410) were collected and 4338 independent reflections were used for structure solution and refinement. The crystal structure solution and refinement were performed using the SHELX-2018 package. Atomic positions were located using direct methods and refined using a combination of Fourier synthesis and least-square refinement in isotropic and anisotropic approximations. The refinement converged to R_1_ = 0.0551 (for 5961 observed reflections), wR_2_ = 0.1436, and GOF = 1.020. A summary of crystallographic data for the single-crystal experiments is available from The Cambridge Crystallographic Data Centre (CCDC) via http://www.ccdc.cam.ac.uk (accessed on 4 May 2023), ref. number 2258627.

### 2.9. Synthesis

#### 2.9.1. Synthesis of *N*,*N*′-Bis(3-methoxyphenyl)-4,6-dinitro-1,3-benzenediamine

1,5-Dichloro-2,4-dinitrobenzene (2.37 g, 0.01 mol) was added in small portions to a solution of *m*-anisidine (2.70 g, 0.022 mol) in DMAc (8 mL) and triethylamine (3 mL) at 60 °C. The mixture was stirred for 8 h, then precipitated with ethanol (100 mL) and filtered to obtain 3.64 g (89%) of the product. Melting point 146–147 °C. Elemental analysis: calc. C 58.54; H 4.42; N 13.65. C_20_H_18_N_4_O_6_ M-410.4. Found C 58.43; H 4.45; N 13.48. ^1^H NMR (400 MHz, DMSO-d_6_) δ 9.03 (s, 1H), 7.26 (t, 2H), 6.88 (m, 4H), 6.77 (dd, 2H), 6.49 (s, 2H), 3.72 (s, 6H) (Figure S1).

#### 2.9.2. Synthesis of *N*^*1*^,*N*^*5*^-Bis(3-methoxyphenyl)-1,2,4,5-benzenetetramine (**1**)

*N^1^*,*N^5^*-bis(3-methoxyphenyl)-4,6-dinitro-1,3-benzenediamine (3.5 g, 8.53 mmol) was mixed with ethanol (100 mL) and hydrogenated in 400 mL autoclave with 10% Pd/C catalyst (0.3 g) for 8 h with a pressure of 80 bar at 80–90 °C. The product solution was passed through silica gel and concentrated to a total volume of 100 mL using a rotary evaporator. Then it was cooled until precipitation of the product. The product was filtered and dried to obtain 2.72 g (91%). Melting point 189–191 °C. Elemental analysis: calc. C 58.54; H 4.42; N 13.65. C_20_H_22_N_4_O_2_. M 350.42. Found C 68.55; H 6.33; N 15.99. ^1^H NMR (400 MHz, DMSO-d_6_) δ 6.94 (t, J = 8.0 Hz, 2H), 6.86 (s, 2H), 6.58 (s, 1H), 6.20–6.08 (m, 7H), 4.47 (s, 4H), 3.62 (s, 6H) (Figure S2).

#### 2.9.3. Synthesis of *N*^*2*^,*N*^*4*^-Bis(*p*-trifluorobenzoyl)-*N*^*1*^,*N*^*5*^-bis(3-methoxyphenyl)-1,2,4,5-benzenetetramine (**2**)

4-(Trifluoromethyl)benzoyl chloride (0.44 g, 2.1 mmol) solution in toluene (0.5 mL) was gradually added to solution of *N*^*1*^,*N*^*5*^-bis(3-methoxyphenyl)-1,2,4,5-benzenetetramine (**1**) (0.35 g, 1 mmol) in NMP (3 mL) and stirred for 2 h at room temperature. The mixture was poured in isopropanol (50 mL), then filtered and dried to obtain 0.66 g of the product (95%). Melting point 201–204 °C. Elemental analysis: calc. C 62.25; H 4.06; F 16.41 N 8.09. C_36_H_28_F_6_N_4_O_4_. M 694.6. Found C 62.33; H 4.16; F 16.31 N 8.04. ^1^H NMR (400 MHz, DMSO-d_6_) δ 9.96 (s, 1H), 8.13 (d, J = 8.1 Hz, 4H), 7.89 (d, J = 8.2 Hz, 4H), 7.70 (s, 1H), 7.57 (s, 1H), 7.23 (s, 1H), 7.10 (t, J = 8.1 Hz, 2H), 6.62–6.55 (m, 2H), 6.51 (s, 2H), 6.38 (dd, J = 8.3, 2.4 Hz, 2H), 3.68 (s, 6H) (Figure S3).

#### 2.9.4. Synthesis of 2,6-Bis(*p*-trifluorophenyl)-1,7-dihydro-1,7-Bis(m-methoxyphenyl)-benzo [1,2-d:4,5-d′]diimidazole (**3**)

0.6 g (0.9 mmol) of *N^2^*,*N^4^*-bis(*p*-trifluorobenzoyl)-*N^1^*,*N^5^*-bis(3-methoxyphenyl)-1,2,4,5-benzenetetramine (**2**) was heated in a test tube in argon flow at 330 °C for 1 h. After cooling, 0.56 g (98%) of the product (**3**) was isolated. Melting point 328–330 °C. Elemental analysis: calc. C 65.65; H 3.67; F 17.31 N 8.51. C_36_H_24_F_6_N_4_O_2_. M 658.6. Found C 65.55; H 3.61; F 17.22, N 8.48.

#### 2.9.5. Synthesis of Polyamide Pre-Polymer (**4**)

[1,1′-Biphenyl]-4,4′-dicarbonyl dichloride (1.1165 g, 0.004 mol) was gradually added to a solution of *N^1^*,*N^5^*-bis(3-methoxyphenyl)-1,2,4,5-benzenetetramine (**1**) (1.6419 g, 0.004 mol) in DMAc (6 mL). The mixture was stirred for 24 h under argon flow. Pre-polymer polyamide films were prepared by casting 10 wt.% polymer reaction solutions onto glass substrates. The films were dried at 60 °C overnight and then peeled off by immersion in water. Part of the solution was precipitated in methanol, filtered and dried to obtain samples for further analyses. Reduced viscosity η_red_ 0.83 dL/g (25°C, 0.5 g/dL, NMP). GPC (NMP): M_n_ 103 kDa, M_w_ 185 kDa (PDI 1.8). ^1^H NMR (Figure S4).

#### 2.9.6. Thermal Heterocyclizaton

Thermal heterocyclizaton of polyamide pre-polymer (**4**) film was carried out in argon flow at 300–350 °C with the formation of PBI-OMe (**5**).

### 2.10. HT-PEM Fuel Cell Operation

Operation of HT-PEMFC membrane-electrode assembly (MEA) with the PBI-OMe membrane was studied using a standard test cell with two graphite flow field plates (Arbin Instruments, College Station, TX, USA) at 160, 180, and 200 °C. Standard gas diffusion electrodes Celtec^®^-P Series 1000 MEA with Pt loadings of 1.0 mgPt/cm^2^ for the anode and 0.75 mgPt/cm^2^ for the cathode [33] (BASF, Ludwigshafen am Rhein, Germany) were used to build the MEA. The MEA was completed by placing the PBI-OMe membrane (~100 µm) between the gas diffusion electrodes during the assembly of the test cell. About 25% contraction of the gas diffusion electrodes was achieved by the use of polytetrafluoroethylene spacers of the required thickness.

The MEA polarization curves were recorded at ambient pressure of 160, 180, and 200 °C. The working area of the membrane-electrode assembly was 5 cm^2^. The anode was supplied with hydrogen obtained by electrolysis from a GVCh-6 hydrogen generator (Khimelektronika, Moscow, Russia) at a rate of 100 mL/min, and the cathode was supplied with atmospheric air at a rate of 800 mL/min without additional humidification with no backpressure. The polarization curves were obtained on a P-150X Potentiostat-galvanostat (Electrochemical Instruments, Chernogolovka, Russia). In voltammetry measurements fuel cell voltage was scanned at the rate of 10 mV/s in the range 0.95–0.15 V, and voltammetry curves stabilized after 2–3 h of the cell voltage cycling.

The HT-PEM fuel cell membrane through plane resistance (mΩ cm^2^) was obtained by the method of electrochemical impedance spectroscopy (EIS). The EIS experiments were performed on a SmartStat PS-250 Potentiostat-galvanostat (Electrochemical Instruments, Chernogolovka, Russia) at 0.4 A/cm^2^, in the frequency range 50 kHz–0.1 Hz.

The upper limit of hydrogen crossover through the membrane at the operating temperatures of the HT-PEM fuel cell (180 °C) was measured by the method of linear sweep voltammetry by supplying hydrogen to the anode and argon (99.998% purity) to the cathode; the gases were supplied at ambient pressure with flow rates of 50 mL/min. Finally, the open-circuit voltage reached a pseudo-steady-state value, and the voltage was swept slowly with a rate of 1 mV/s to 500 mV; the current of hydrogen oxidation was recorded. Hydrogen penetrated the membrane, then appeared in the cathode catalyst layer and was completely oxidized, so that, the current value measured under these conditions is assumed to be equal to the flow of hydrogen diffusing through the membrane. The current density corresponding to the crossover of hydrogen was calculated at 350 mV, and higher electrode potentials were avoided to prevent platinum oxidation.

### 2.11. Electrochemical Impedance Spectroscopy

The conductivity measurements of through-plane proton conductivity for the PBI-OMe and PBI-OP membranes were performed by electrochemical impedance spectroscopy (EIS) on an Elins Z500-PRO impedance meter (Chernogolovka, Russia) in the range of frequencies 10^–2^–10^6^ Hz in the potentiostatic mode with 80 mV sinusoidal excitation voltage in a two-electrode cell with graphite electrodes. The conductivity values were obtained for temperatures 25–180 °C with a step of 10 °C. The values of proton conductivity were taken at the intercept of the semicircle of the Nyquist plot with the real impedance axis in the high-frequency region.

The proton conductivity [34] (*σ*, S/cm) was calculated according to Equation (1)

(1) $$\sigma =\frac{h}{Sr}$$

where *h* is the membrane thickness, *S* is the electrode area and *r* is measured resistance.

The activation energy of conductivity (*E_a_*) was calculated graphically from the dependence of the logarithm of conductivity log *σ* vs. 1000/*T* using the Arrhenius Equation (2) [35] for the linear part of the curve at *T* > 80 °C.

*σ* = *σ*_0_ exp (−*E_a_*/*RT*) (2)

where *T* is temperature and *R* is universal gas constant

## 3. Results and Discussion

**Figure 3:**
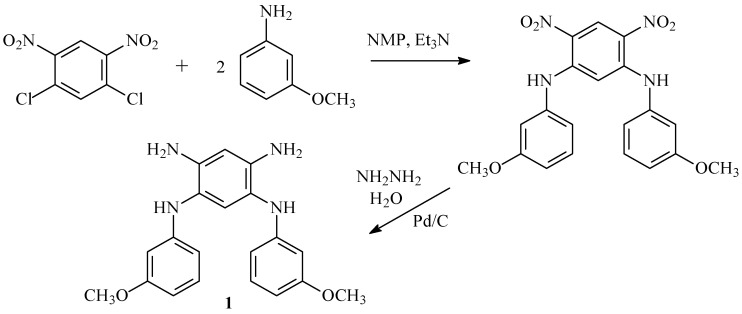
Synthesis of *N^1^*,*N^5^*-bis(3-methoxyphenyl)-1,2,4,5-benzenetetramine (**1**).

To verify the proposed assumption, a new monomer, *N^1^*,*N^5^*-bis(3-methoxyphenyl)-1,2,4,5-benzenetetramine (**1**), was synthesized according to the scheme (Figure 3). Additional experimental results are provided in Figures S1 and S2.

**Figure 4:**
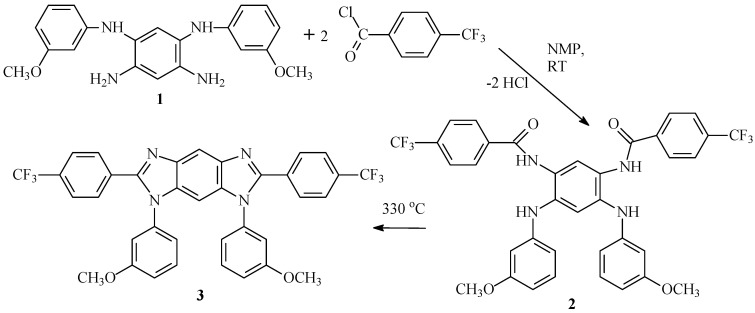
A two-stage model reaction.

Before starting the synthesis of polymers, a two-stage model reaction was carried out (Figure 4).

**Figure 5:**
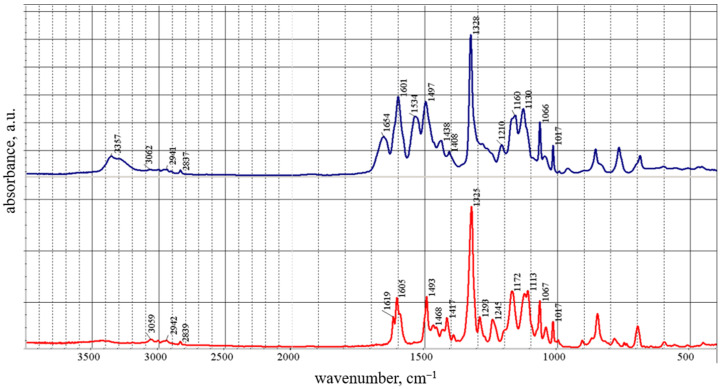
FTIR spectra of **2** (**top**, blue) and **3** (**bottom**, red) in KBr pellet.

The structures of compounds **1**–**3** were confirmed by NMR, FTIR spectroscopy, and elemental analysis (Figures S2 and S3). Thus, during the thermal cyclization of **2** in **3**, significant changes occurred in the FTIR spectrum of **3** (Figure 5).

The spectrum of **3** completely lacks bands of amide I (1654 cm^−1^) and amide II (1534 cm^−1^), as well as bands in the region of 3200–3400 cm^−1^ belonging to the –NH-groups of compound **2** [36].

**Figure 6:**
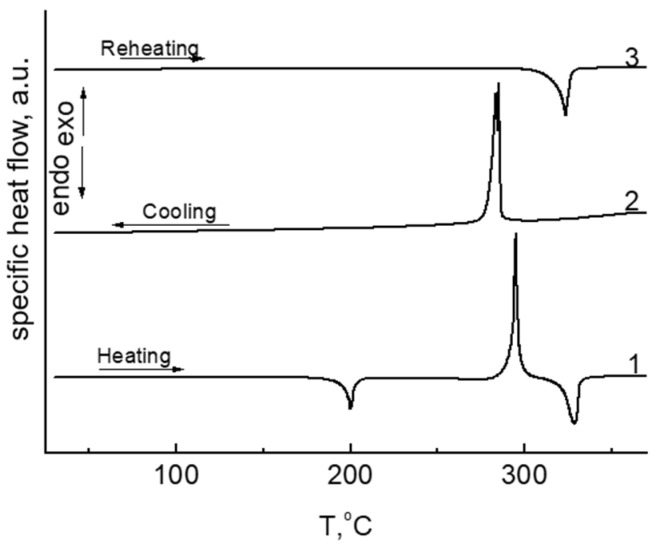
DSC traces for compound **2** and product of its cyclization **3**: for heating (**bottom**), cooling (**central**), and reheating (**top**) at a heating/cooling rate of 10 K/min in argon.

Figure 6 shows the results of the DSC confirming the cyclization of compound **2** at elevated temperatures.

**Figure 7:**
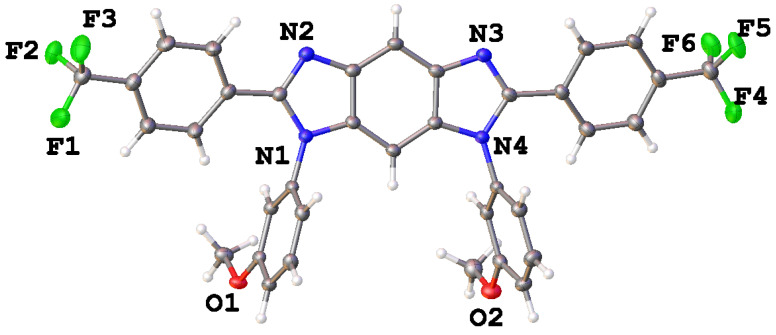
General view of **3** in the representation of atoms by thermal ellipsoids (*p* = 50%).

The structure of compound **3** was also confirmed by X-ray diffraction analysis (Figure 7).

The trifluoromethyl-substituted rings are almost coplanar to the central heterocycle with CCNC torsion angles equal to 14 and 16°. In contrast, the methoxy-substituted rings are located perpendicular to the central part with dihedral angles ca. 95°. Such mutual orientation of aromatic rings almost excludes the possibility of the formation of intramolecular stacking interactions. As a consequence, the molecules in the crystal are assembled due to weak π-interactions such as C-H···O; C-H···F and C-H···N.

**Figure 8:**
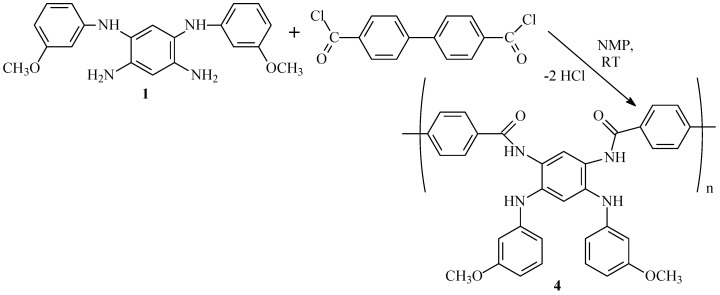
Polyamide synthesis from tetraamine and dicarbonyl dichloride.

At the next stage of the research, the polycondensation of [*N^1^*,*N^5^*-bis(3-methoxyphenyl)-1,2,4,5-benzenetetramine] (**1**) with [1,1′-biphenyl]-4,4′-dicarbonyl dichloride was investigated. The reaction proceeds smoothly in DMAc or NMP medium at room temperature with a total monomer concentration of 35 wt.% to form a high-molecular-weight film-forming polyamide (**4**) with side methoxy-substituents (Figure 8).

**Figure 9:**
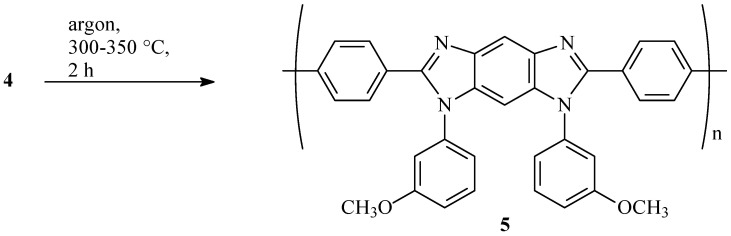
Thermal heterocyclization of polyamide **4**.

Additional experimental results for **4** are provided in Figure S4. According to GPC (Figure S5), its molecular weight reaches M_w_ 185 kDa, M_n_ 103 kDa (PDI 1.8). Films cast from reaction mixtures (tensile strength, σ, 80–90 MPa; elongation at break, ε, 8–12%; Young’s modulus, E, 1700–1900 MPa) were introduced to thermal heterocyclization in argon at 300–350 °C for 2 h to form PBI-MeO functionalized with methoxy groups (**5**) (Figure 9).

PBI-OMe films after thermal heterocyclization possess sufficient tensile strength, σ, 70–80 MPa; elongation at break, ε, 6–10%; and Young’s modulus, E, 2000–2200 MPa, which determines the possibility of their further use as a matrix for proton-conducting membranes doped with phosphoric acid.

**Figure 10:**
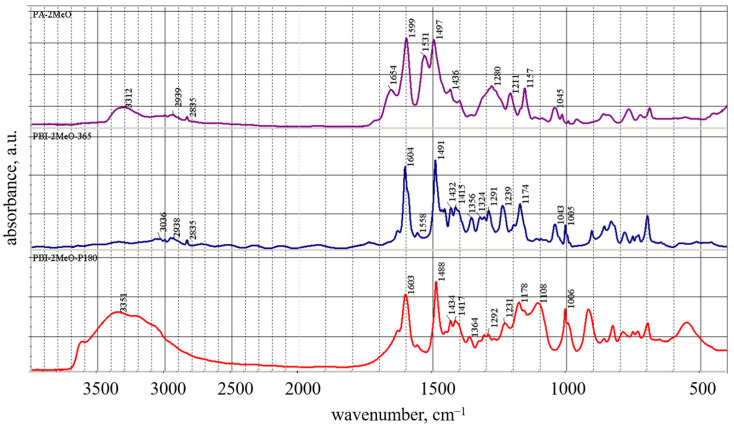
FTIR spectra of polyamide (**4**) (violet), PBI-MeO (**5**) (blue), and **5** immersed in H_3_PO_4_ (PBI-OP, **6**) (red).

The process of thermal heterocyclization of polyamide (**4**) into PBI-MeO (**5**) was controlled by FTIR-spectroscopy (Figure 10).

The absorption bands of amide groups such as amide I (1654 cm^−1^) and amide II (1531 cm^−1^), as well as bands in the region 3200–3400 cm^−1^ which belong to the –NH-groups, completely disappeared, as was shown by the example of the model reactions given above. After heating **5** in 85% phosphoric acid for 6 h at 180 °C, the disappearance of methoxy-groups in the region 2800–2900 cm^−1^ and the appearance of three characteristic absorption maxima in the areas of 1108 cm^−1^ (HPO_4_^2−^), 990 cm^−1^ (P-OH), and 930 cm^−1^ (H_2_PO_4_^−^) and large absorption region at 2500–3500 cm^−1^ (-OH) were observed (PBI-OP, **6**, Figure 10). A peak at ~520 cm^−1^ is a feature of phosphoric acid-doped PBI [37].

According to elemental analysis, the phosphorus content in phosphorylated PBI-OP (**6**) after washing with water until the neutral pH of water was 10.35% (calc. 9.46), which clearly indicates the complete substitution of methoxy-groups by phosphoric acid.

It is important to note that after thermal heterocyclization of the polyamide film into the PBI-OMe, the polymer completely loses its solubility in phosphoric and sulfuric acids, which makes it possible to avoid the necessary and time-consuming procedure of film crosslinking for almost all PBI. The crosslinking of PBIs allows the saving of strength properties of the membranes in the phosphoric acid-doped state at high temperatures (150–200 °C) [30,38].

**Figure 11:**
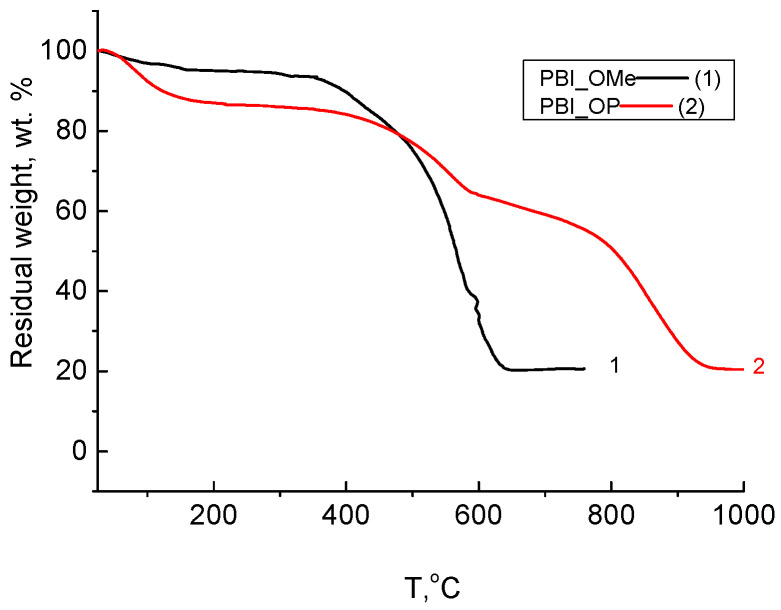
TGA curves (air) for PBI-OMe (**5**) after cyclization of (black) and PBI-OP (**6**) (treated by phosphoric acid at 180 °C) (red).

PBI-OMe (**5**) and PBI-OP (**6**) were also investigated by TGA in air (Figure 11).

According to the TGA data in air (Figure 11), it follows that PBI-OMe (**5**) has very high thermal characteristics, starting to decompose in air above 400 °C. Its phosphorylated form demonstrates even higher thermal stability, and PBI-OP (**6**) starts to decompose above 500 °C.

At the next stage of research, attention was focused on doping the PBI-OMe films with 85% phosphoric acid in order to obtain proton-conducting membranes and test them as part of a membrane-electrode assembly in the HT-PEM fuel cell.

The PBI-OMe film was doped by phosphoric acid (85 wt.%) at 60 °C for 120 h to a three-fold increase in weight which corresponds to ~10 phosphoric acid molecules per polymer molecular unit.

**Figure 12:**
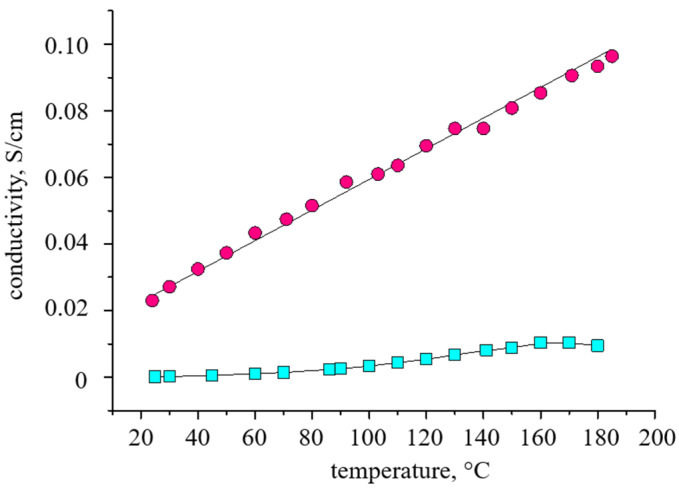
Temperature dependence of the proton conductivity for the PBI-OMe (squares) and PBI-OP (circles) membranes.

It is quite surprising that the proton conductivity of the doped PBI-OMe membrane (Figure 12) showed low values, but increased slightly with temperature up to 5.4 mS/cm at 180 °C.

**Figure 13:**
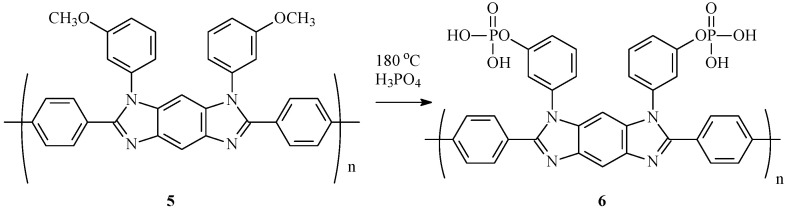
Phosphorylation of PBI-OMe (**5**) with the formation of PBI-OP (**6**).

The activation energy of conductivity (Figures S6 and S7) is 27.6 ± 0.6 kJ/mol. The proton conductivity of the membrane after conditioning at 180 °C for 6 h increases significantly, reaching 96 mS/cm at 185 °C. The activation energy in this case is found to be 8.0 ± 0.2 kJ/mol. Such changes in the proton conductivity can be explained by a change in the chemical composition of the polymer due to the replacement of methoxy-groups in **5** (Figure 13). A similar increase in proton conductivity was observed by the authors of [39] for sulfonated PBI, however, because of different reasons.

**Figure 14:**
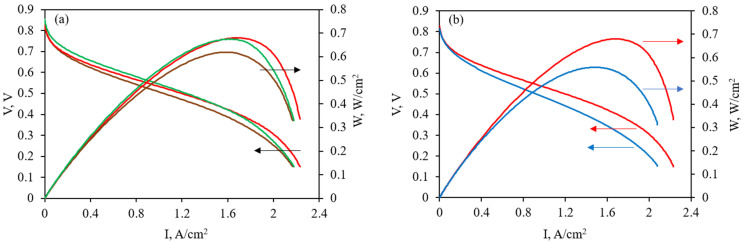
Operation of hydrogen/air HT-PEMFC MEA: (**a**) polarization and power density curves for the PBI-OMe/PBI-OP membrane at 160 °C (brown), 180 °C (red), and 200 °C (green); (**b**) comparison of polarization and power density curves at 180 °C for the PBI-OMe/PBI-OP (red) and Celazole^®^ (blue) membranes.

The length and width changes were no more than 5%, the thickness increased by 10% (by ~10 µm). High proton conductivity and excellent mechanical properties of the PBI-OMe/PBI-OP membrane allowed it to be tested in the HT-PEMFC MEA with Celtec^®^ P1000 commercial electrodes. MEA polarization and power density curves at 160–200 °C are shown in Figure 14a.

Stable MEA operation at these temperatures was observed for ~300 h (13 days). The highest power density reached 680 mW/cm^2^ with low membrane resistance of 49.6 mΩ cm^2^ at MEA operation conditions of 180 °C according to EIS data. The upper limit of the electrochemical hydrogen crossover through the membrane was found to be 3.4 mA/cm^2^ according to linear sweep voltammetry. This value was lower than 4–5 mA/cm^2^ for the Celazole^®^ membrane [40] and was similar to the PBI-OPhT membrane (~3 mA/cm^2^) [30]. A comparison of the PBI-OMe/PBI-OP membrane with the commercially available Celazole^®^ membrane when operated in MEA is shown in Figure 14b. The data from polarization and power density curves indicate better performance of the PBI-OMe/PBI-OP membrane in the HT-PEMFC MEA.

## 4. Conclusions

It was shown for the first time that polybenzimidazoles containing methoxy- substituents (PBI-OMe) in the side chain, doped with phosphoric acid, undergo self-phosphorylation at operation conditions for a hydrogen/air HT-PEM fuel cell. The proton conductivity of such a membrane increases by one order of magnitude and reaches 100 mS/cm at 180 °C. The membrane-electrode assembly with the PBI-OMe/PBI-OP membrane and Celtec^®^-P1000 electrodes exceeds the commercial analog with the Celazol^®^ membrane in terms of power characteristics. The achieved peak power was 680 mW/cm^2^ at 180 °C. The two-stage synthesis of the new PBI through the stage of obtaining the fore-polyamide (pre-polymer) at room temperature, followed by the thermal heterocyclization, allows moving toward the elimination of environmental problems related to the production of industrial PBIs, and sharply reducing the cost of proton-conducting membranes for hydrogen/air HT-PEM fuel cells.

## Data Availability

The authors confirm that the data supporting the findings of this study are available within the article and its Supplementary Materials.

## Supplementary Materials

The following supporting information can be downloaded at: https://www.mdpi.com/article/10.3390/membranes13060552/s1, Figure S1: ^1^H NMR of *N*′-bis(3-methoxyphenyl)-4,6-dinitro-1,3-benzenediamine; Figure S2: ^1^H NMR of *N^1^*,*N^5^*-bis(3-methoxyphenyl)-1,2,4,5-benzenetetramine (1); Figure S3: ^1^H NMR of *N^2^*,*N^4^*-bis(*p*-trifluorobenzoyl)-*N^1^*,*N^5^*-bis(3-methoxyphenyl)-1,2,4,5-benzenetetramine (2); Figure S4: ^1^H NMR of polyamide (4); Figure S5: GPC of polyamide (4) in NMP; Figure S6. Arrhenius plot for the PBI-OMe; Figure S7. Arrhenius plot for the PBI-OP.
