# Supplementary material for: Self-Phosphorylated Polybenzimidazole: An Environmentally Friendly and Economical Approach for Hydrogen/Air High-Temperature Polymer-Electrolyte Membrane Fuel Cells
Source: Membranes (Basel). 2023 May 25;13(6):552. doi: 10.3390/membranes13060552 (PMC10303732; doi:10.3390/membranes13060552)
Supplement: Supplementary file 1 [file membranes-13-00552-s001.zip › membranes-2409950-supplementary.pdf]

Supplementary Materials

N′-bis(3-methoxyphenyl)-4,6-dinitro-1,3-benzenediamine

Melting point (m.p.) 146–147 °C. Elemental analysis: calc. C 58.54; H 4.42; N 13.65. $C_{20}H_{18}N_4O_6$ M-410.4. Found C 58.43; H 4.45; N 13.48.

^{1}H NMR (400 MHz, DMSO-d_6) δ 9.03 (s, 1H), 7.26 (t, 2H), 6.88 (m, 4H), 6.77 (dd, 2H), 6.49 (s, 2H), 3.72 (s, 6H)

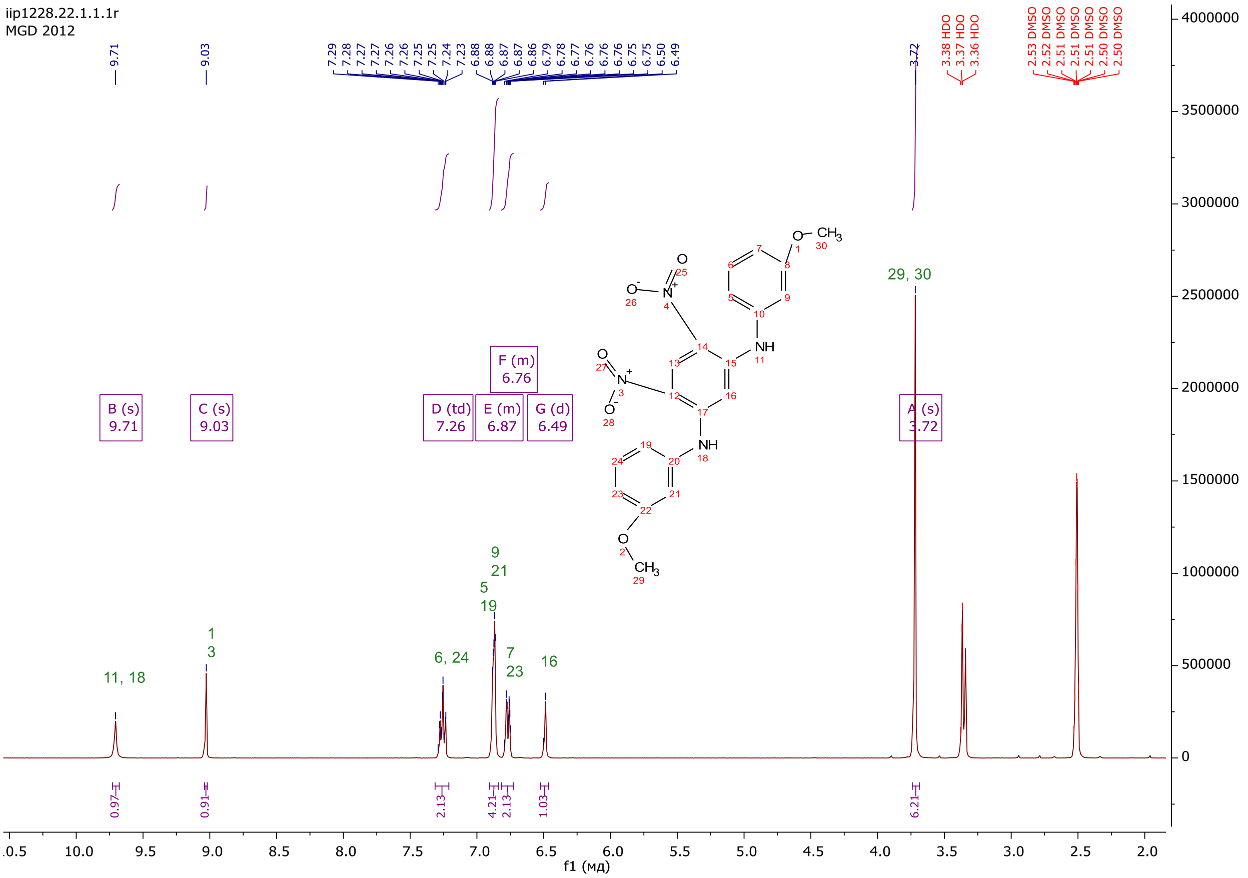

Figure S1. ^{1}H NMR of *N′*-bis(3-methoxyphenyl)-4,6-dinitro-1,3-benzenediamine.

N[1],N[5]-bis(3-methoxyphenyl)-1,2,4,5-benzenetetramine (**1**)

Melting point (m.p.) 189-191 °C. Elemental analysis: calc. C 58.54; H 4.42; N 13.65. $C_{20}H_{22}N_4O_2$. M 350.42. Found C 68.55; H 6.33; N 15.99.

^{1}H NMR (400 MHz, DMSO-d_6) δ 6.94 (t, J = 8.0 Hz, 2H), 6.86 (s, 2H), 6.58 (s, 1H), 6.20 – 6.08 (m, 7H), 4.47 (s, 4H), 3.62 (s, 6H).

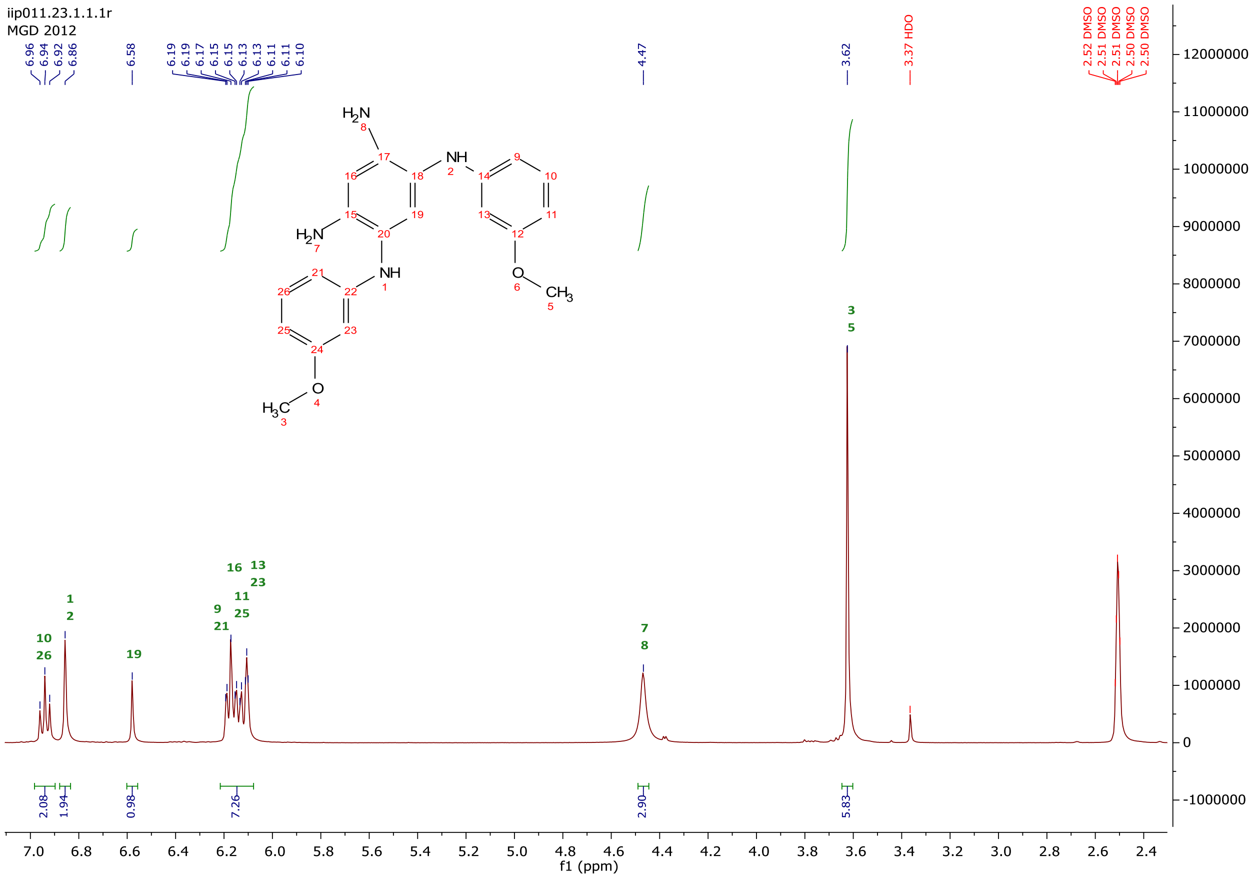

Figure S2. ^{1}H NMR of N^1,N^5-bis(3-methoxyphenyl)-1,2,4,5-benzenetetramine (**1**).

N^2,N^4-bis(*p*-trifluorobenzoyl)-N^1,N^5-bis(3-methoxyphenyl)-1,2,4,5-benzenetetramine (**2**)

Melting point (m.p.) 201-204 °C. Elemental analysis: calc. C 62.25; H 4.06; F 16.41 N 8.09. C36H28F6N4O4. M 694.6. Found C 62.33; H 4.16; F 16.31 N 8.04.

^{1}H NMR (400 MHz, DMSO-d_6) δ 9.96 (s, 1H), 8.13 (d, *J* = 8.1 Hz, 4H), 7.89 (d, *J* = 8.2 Hz, 4H), 7.70 (s, 1H), 7.57 (s, 1H), 7.23 (s, 1H), 7.10 (t, *J* = 8.1 Hz, 2H), 6.62 – 6.55 (m, 2H), 6.51 (s, 2H), 6.38 (dd, *J* = 8.3, 2.4 Hz, 2H), 3.68 (s, 6H).

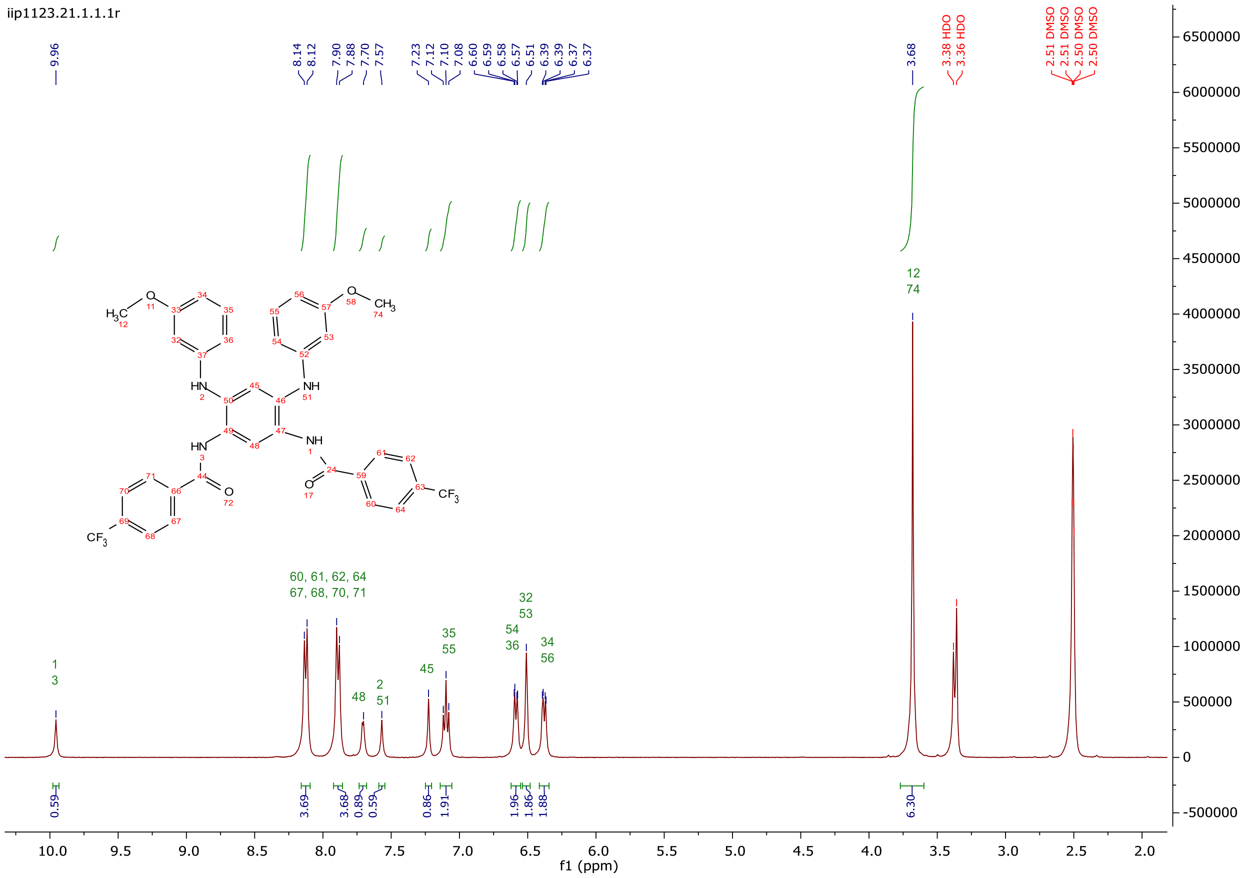

Figure S3. ^{1}H NMR of N^2,N^4-bis(*p*-trifluorobenzoyl)-N^1,N^5-bis(3-methoxyphenyl)-1,2,4,5-benzenetetramine (**2**).

Reduced viscosity η_{red} 0.83 dL/g (25°C, 0.5 g/dL, NMP). GPC (NMP): M_n 103 kDa, M_w 185 kDa (PDI 1.8).

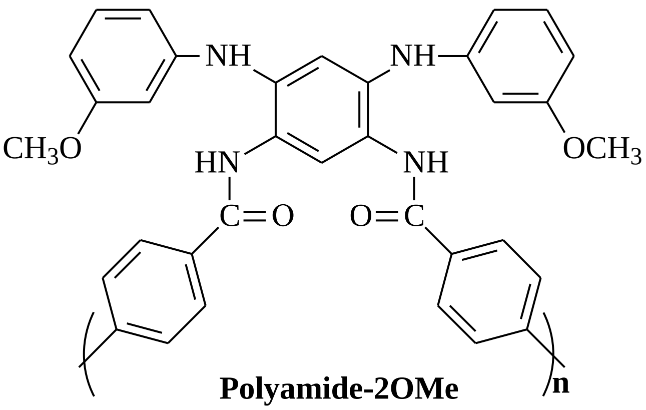

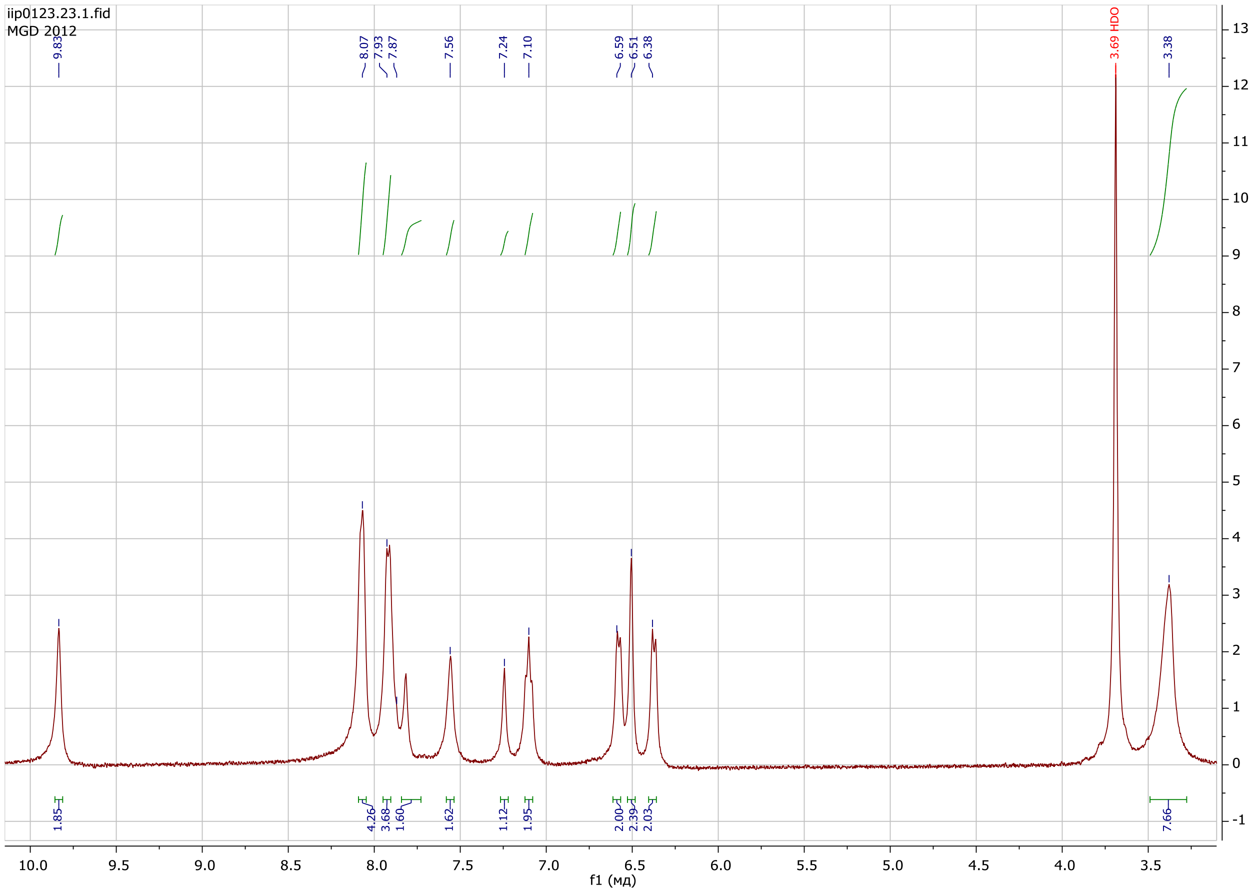

Figure S4. ^{1}H NMR of polyamide (**4**).

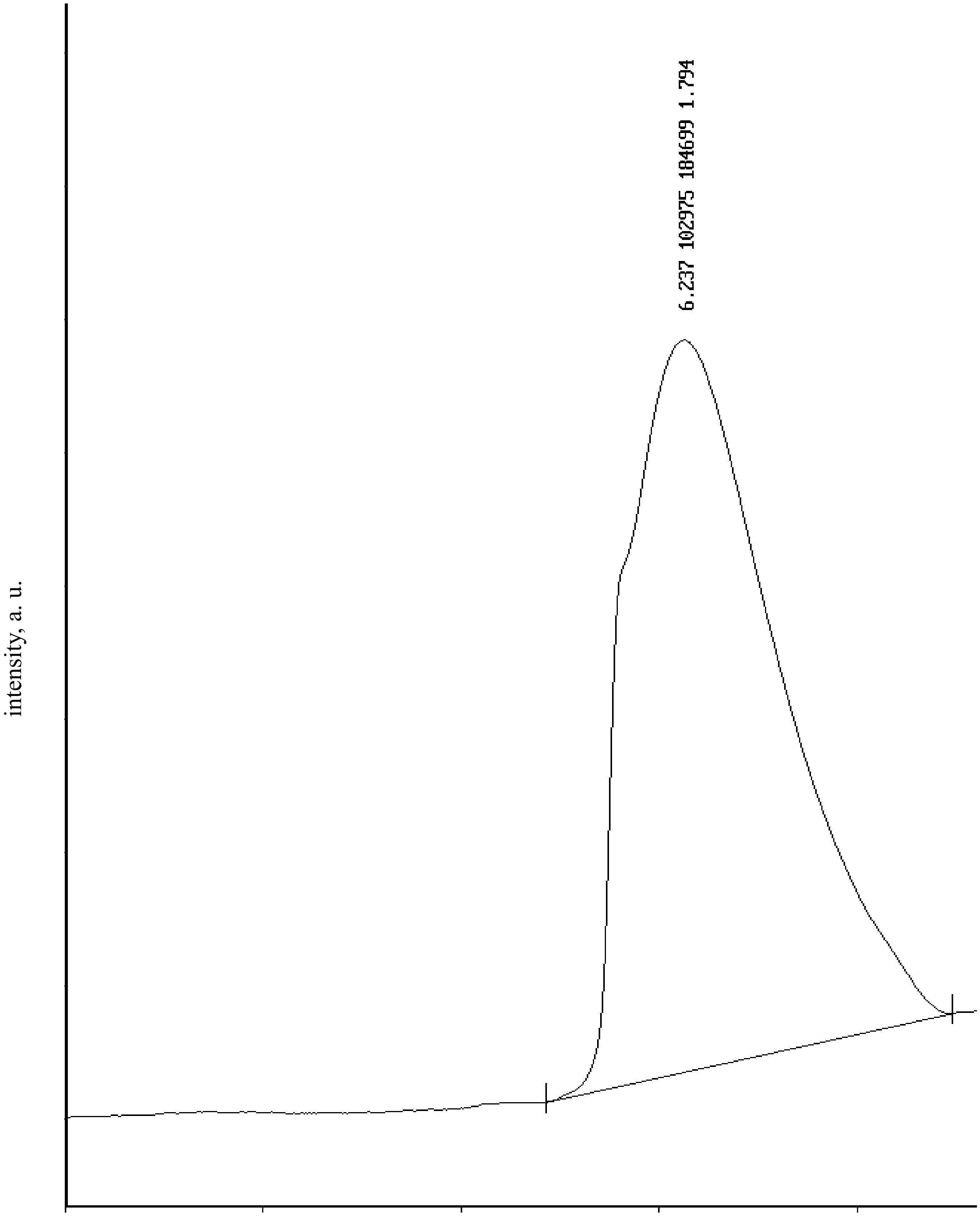

Figure S5. GPC of polyamide (**4**) in NMP.

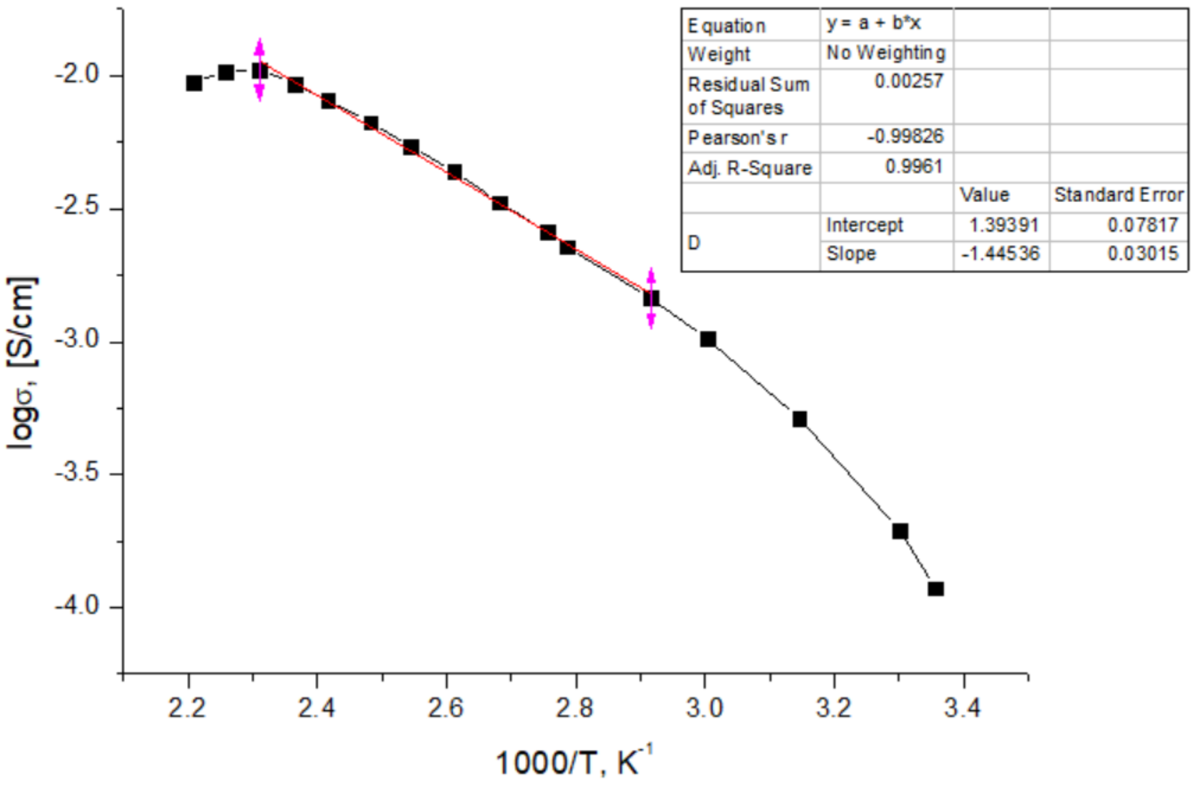

Figure S6. Arrhenius plot for PBI-OMe.

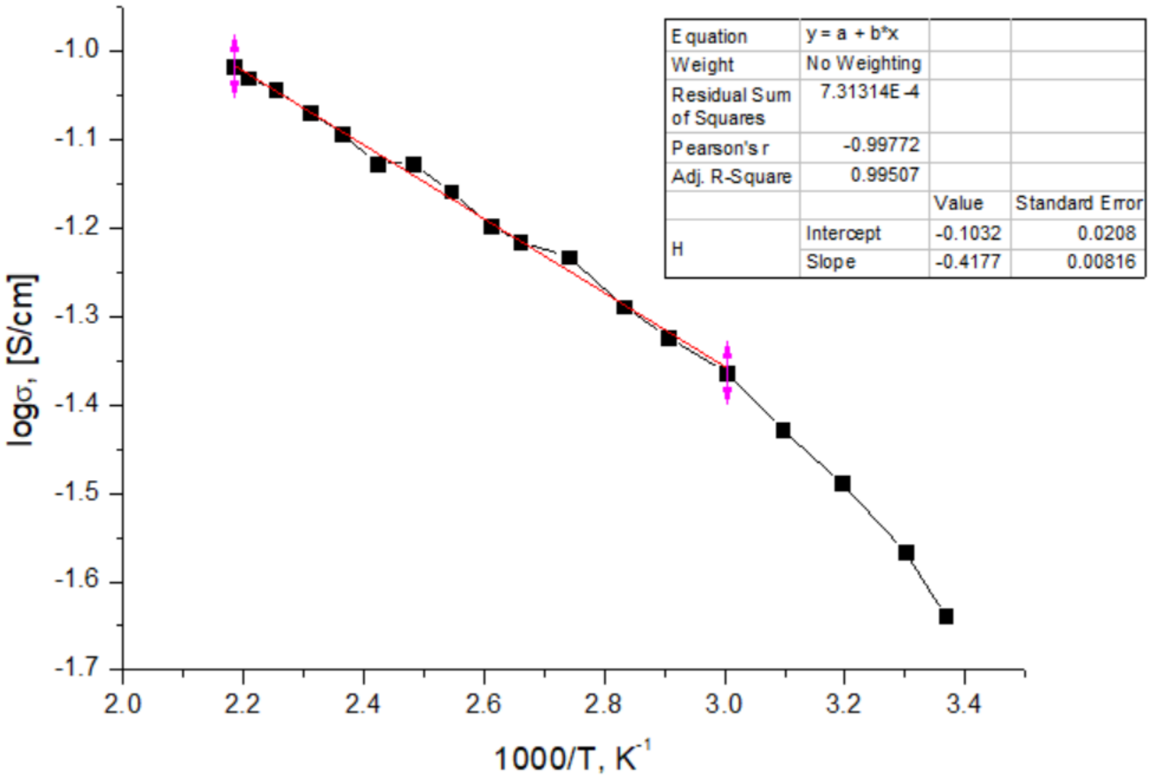

Figure S7. Arrhenius plot for PBI-OP.
